# The Frequency and Spread of a GABA-Gated Chloride Channel Target-Site Mutation and Its Impact on the Efficacy of Ethiprole Against Neotropical Brown Stink Bug, *Euschistus heros* (Hemiptera: Pentatomidae)

**DOI:** 10.3390/insects16040422

**Published:** 2025-04-17

**Authors:** Ana C. P. Cuenca, Bettina Lueke, Renato Vicentini, Ralf Nauen

**Affiliations:** 1 Bayer S.A., Crop Science, São Paulo 13148-914, Brazil; ana.cuenca@bayer.com; 2 Systems Biology Laboratory, Institute of Biology, University of Campinas, São Paulo 13083-872, Brazil; 3 Bayer AG, Crop Science Division, R&D, 40789 Monheim, Germany; bettina.lueke@bayer.com

**Keywords:** *Euschistus heros*, neotropical brown stink bug, soybean, phenylpyrazole, ethiprole, A301S mutation, target-site resistance, RDL

## Abstract

The Neotropical brown stink bug (NBSB), *Euschistus heros*, is the most prevalent sucking soybean pest in Brazil, and control of it largely relies on the application of synthetic insecticides such as ethiprole, a phenylpyrazole insecticide targeting GABA-gated chloride channels encoded by the *Rdl* (resistant to dieldrin) gene. This study monitored 41 NBSB populations collected between 2021 and 2024 and revealed, for the first time, the presence of a mutation, A301S, in NBSB RDL receptors commonly known to confer target-site resistance to channel blockers such as phenylpyrazoles. Laboratory contact bioassays with ethiprole at 150 g a.i./ha (ethiprole label dose) revealed that most populations were quite susceptible, despite rather high resistance allele frequencies in some populations. Genotyping results confirmed that susceptible and A301S heterozygous genotypes largely dominate in frequency compared to homozygous resistant individuals, which exhibited high survivorship (84%) when exposed to discriminating rates of ethiprole in laboratory bioassays, while susceptible and heterozygote individuals showed lower survival rates (13% and 34%, respectively), suggesting an incompletely recessive trait conferring ethiprole resistance. Furthermore, we developed a TaqMan assay for molecular genotyping to monitor the spread of resistance allele frequency and to inform resistance management strategies for sustainable NBSB control using highly effective phenylpyrazole insecticides such as ethiprole.

## Simple Summary

The Neotropical brown stink bug (NBSB) is the most common sucking soybean pest in Brazil. One of the most recent insecticides introduced to control this pest is ethiprole, a phenylpyrazole targeting GABA-gated chloride channels in the insect nervous system. This study monitored 41 NBSB populations from 2021 to 2024 and revealed the presence of a mutation, A301S, in GABA-gated chloride channels known to confer resistance to channel blockers such as phenylpyrazole insecticides. Adult vial bioassays revealed that most populations were quite susceptible to ethiprole at recommended label rates, despite rather high resistance allele frequencies in some populations. This is because susceptible and A301S heterozygous genotypes largely dominate in frequency compared to homozygous resistant individuals, which showed high survivorship (84%) when exposed to discriminating rates of ethiprole in laboratory bioassays, while susceptible and heterozygote individuals showed lower survival rates (13% and 34%, respectively), suggesting an incompletely recessive trait conferring ethiprole resistance in NBSBs. The results obtained in this study will help in the effective implementation of resistance management strategies for sustainable NBSB control and in extending the lifecycle of phenylpyrazole insecticides such as ethiprole.

## 1. Introduction

The Neotropical brown stink bug (NBSB), *Euschistus heros* Fabricius, 1798 (Hemiptera: Pentatomidae), is an important hemipteran pest in Brazil widely distributed in agricultural production areas [1], commonly attacking soybean (*Glycine max* L. (Merr.)), corn (*Zea mays* L.), cotton (*Gossypium hirsutum* L.) and sunflower (*Helianthus annuus* L.) plants [2,3]. The NBSB is native to the Neotropical region and is commonly found in Brazil as well as neighboring countries including Argentina [4], Uruguay and Paraguay [5,6].

The NBSB feeds on branches or stems of the pods of the soybean plant by inserting its mouth parts into the vascular system, injecting salivary secretions that promote the retention of leaves in vegetative stages, leading to pod and seed malformation, abscission, the darkening of seeds, delayed seed maturation and decreased germination and seedling vigor [7,8]. The NBSB has a major impact on soybean crops and can cause pod abortion and a reduction in grain weight, compromising grain quality and productivity by up to 30% [9]. In cotton crops, lesions caused by *E. heros* in bolls also result in reductions in fiber quality [2].

*E. heros* management in South American countries largely relies on frequent foliar applications of chemical insecticides. The limited number of chemical classes of insecticides and the repeated and frequent use of the same mode of action (MoA) favor the selection of resistant individuals [9,10], if insect resistance management (IRM) strategies are neglected. Insecticide resistance of *E. heros* has been reported to endosulfan (cyclodienes), monocrotophos and methamidophos (organophosphates) in Brazil [11,12]. Control failures in the field have also been observed after the application of pyrethroids and neonicotinoids, which are frequently used and combined for NBSB control [13,14,15].

Ethiprole is a phenylpyrazole (fiprole) insecticide recently registered for the control of NBSB in Brazil [16]. It belongs to group 2B of the Insecticide Resistance Action Committee (IRAC) MoA classification: GABA (gamma-aminobutyric acid)-gated chloride channel blockers [17]. GABA receptors are widely distributed throughout the insect nervous system [18,19,20] and are important targets of several classes of insecticides, such as cyclodienes, phenylpyrazoles, meta-diamides and isoxazolines [21].

The GABA (RDL) receptor, encoded by the *Rdl* gene (resistance to dieldrin) in many insects, is a homopentameric cys-loop ligand-gated ion channel mediating inhibitory synaptic transmission in the nervous system [20,22]. Several GABA receptor mutations such as R299Q, A301G/N/S, R340Q, T350M and Q359E have been identified in different invertebrate pest species, causing various levels of resistance to different non-competitive channel blockers, including fiproles such as ethiprole [23,24,25,26,27,28]. The A301S mutation, located in the M2 transmembrane region, is the most common substitution found in RDL-GABA receptors, affecting non-competitive channel blocker binding to various degrees [26]. This mutation was first identified in *Drosophila melanogaster*, causing 4000-fold resistance to dieldrin [23] and moderate resistance to phenylpyrazoles [29]. Recently, the A301S mutation was also identified in the brown planthopper *Nilaparvata lugens* and correlated with low levels of fipronil resistance but significant resistance to ethiprole [27,30]. In vitro, the effects of this mutation were electrophysiologically verified by the recombinant expression of *N. lugens Rdl* in Xenopus oocytes (both wildtype and A301S mutant), proving that this mutation has a significant impact on ethiprole binding, whereas it hardly interferes with fipronil binding [27].

Indeed, the evolution of the resistance of *E. heros* to insecticides of various chemical classes poses a significant threat to integrated pest management programs for soybeans in Brazil [31,32,33]. In the present study, we investigated the potential risk of resistance to ethiprole by conducting laboratory bioassays and molecular genotyping studies with field populations of *E. heros* collected in Brazil during three consecutive soybean seasons (2021–2024). For a better understanding of the impact of the RDL-A301S mutation on ethiprole efficacy in NBSBs, we genotyped survivors of discriminating dose bioassays for the presence of the mutation. Based on the data collected, we developed a TaqMan *Rdl*-A301S genotyping assay for future resistance monitoring campaigns to support ethiprole resistance management strategies for sustainable NBSB control in Brazil.

## 2. Materials and Methods

### 2.1. Brown Stink Bug Sampling and Rearing

**Figure 1:**
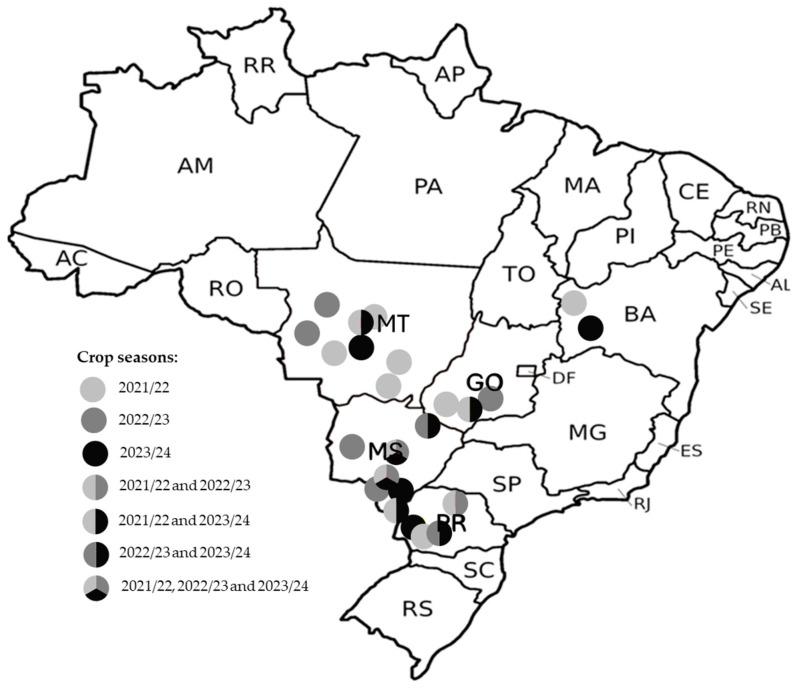
Sampling sites of Neotropical brown stink bugs collected from soybean throughout 2021/22, 2022/23 and 2023/24 soybean seasons in Brazil.

*E. heros* populations were collected in soybean-growing areas of the Bahia, Goiás, Mato Grosso, Mato Grosso do Sul and Paraná states in Brazil throughout the 2021/22, 2022/23 and 2023/24 crop seasons (Figure 1, Table S1). Stink bug populations were then transported to the laboratory (Bayer Field Station, Paulínia, Brazil) in plastic containers (30 cm long × 20 cm wide × 13 cm high) and were fed fresh green bean pods (*Phaseolus vulgaris* L.) and a mix of sunflower seeds (*Helianthus annuus* L.), soybean (*Glycine max* (L.) Merr.) and peanut (*Arachis hypogaea* L.). A small plastic cup containing a piece of cotton moistened with tap water was placed in each container. The insects were kept in a climate-controlled room at 28 ± 1 °C, 60 ± 10% relative humidity and a 12:12 h photoperiod for at least 48 h before bioassays. A *E. heros* population that has been maintained in the laboratory since 2013 without selection pressure by insecticides was used as a susceptible reference strain (Sus).

### 2.2. Phenotypic Monitoring of E. heros Susceptibility to Ethiprole

Vial test (contact) bioassays were performed with a commercial dose (150 g a.i./ha) of ethiprole (Curbix^®^, Bayer Crop Science, São Paulo, Brazil). The technical product (powder, ≥96.5% a.i.) was diluted in acetone (99.5% purity; Sigma-Aldrich, São Paulo, SP, Brazil) using a magnetic stirrer for the complete homogenization of the solution. Next, the inner surface of glass vials (1 cm in diameter) was coated with 0.5 mL of the solution corresponding to the label dose of 150 g a.i./ha (7.5 µg a.i./cm^2^) using a roller shaker (Kasvi, model K45-8010, Sao Paulo, Brazil) at room temperature until the acetone had completely evaporated. The negative control contained only acetone. Adult stink bugs were added to each vial (4 bugs × 5 replicates per concentration), which was partially closed with a lid. Mortality assessment was performed after 24 h of continuous exposure to ethiprole. The insects were scored either alive or dead (no movement when touched with a brush). After scoring, dead and alive insects were separately placed in 15 mL Falcon tubes containing 70% ethanol and stored at−20 °C for later DNA extraction and molecular analysis.

### 2.3. DNA Extraction

Genomic DNA was isolated according to [27] with some modifications: 4 to 6 legs were collected from each insect and were placed in 2 mL plastic tubes, kept in liquid nitrogen for 10 s and transferred to the Tissue Lyser (Qiagen, Hilden, Germany) for maceration for 2 min at 30 Hz, using 2.3 mm metal beads in each tube. Next, 400 µL of 2% cetrimonium bromide was added, and the tubes were vortexed for 10 s, with subsequent incubation for 30 min at 65 °C while shaking (700 rpm). The tubes were centrifuged for 5 min at 14,000× *g*, and the supernatant was transferred to a new 2 mL plastic tube, where 500 µL of chloroform/isoamyl alcohol (24:1) was added and the mixture was shaken by inversion and subsequently centrifuged at 14,000× *g* for 20 min. The supernatant was transferred to new 1.5 mL tubes and, after the addition of 200 µL of ice-cold isopropanol tubes, were incubated at −4 °C overnight. After this period, the tubes were centrifuged at 12,000× *g* for 20 min and the supernatant was discarded. The precipitate was washed with 200 µL of absolute ethanol, followed by another wash with 200 µL of 70% ethanol. The supernatant was discarded, and the pellet was dried at room temperature for 4 h. The DNA was suspended in 40 µL of ultrapure water (ThermoFisher, Waltham, MA, USA). DNA quality was analyzed by Qiaxcel Advanced capillary electrophoresis (Qiagen, Hilden, Germany), and DNA was quantified by spectrophotometry (NanoDrop^®^ One, ThermoFisher Scientific, Waltham, MA, USA). Samples were diluted with ultrapure water to obtain a final concentration of 50 ng/µL.

### 2.4. Partial Sequencing of the RDL-GABA-Gated Chloride Channel in E. heros

The *E. heros* genome available at the National Center for Biotechnology Information (NCBI) under the BioProject accession number PRJNA489772 was utilized for sequence queries. Primers (Table 1) were designed using Geneious Prime software v.2023.2.1 (Biomatters Ltd., Auckland, New Zealand). PCR was performed using 1 μL of DNA (50 ng/μL), 2.5 μL of 5× GoTaq^®^ DNA Polymerase (Promega, Madison, WI, USA), 1 μL of 25 mM MgCl_2_ (Promega, Madison, WI, USA), 0.7 μL of 10 μM primers (forward and reverse), 0.1 μL of GoTaq^®^ DNA Polymerase (5 U/μL, Promega, Madison, WI, USA) and nuclease-free water to a final reaction volume of 12.5 μL. The amplification reactions were conducted with an initial cycle at 95 °C for 2 min, 40 successive cycles composed of three steps (denaturation at 95 °C for 30 s, hybridization at 58 °C for 30 s and synthesis at 72 °C for 30 s) and a final cycle at 72 °C for 5 min. The efficiency of the amplification reaction was verified via electrophoresis in 1.5% agarose gel 7 μL ethidium bromide, in a Tris–acetate–EDTA (TAE) buffer solution (40 mM Tris–acetate, 1 mM EDTA, pH 7.2) at a constant voltage of 100 V and subsequent visualization in a transilluminator. Samples were purified with ExoSAP-IT™ (Applied Biosystems™, Waltham, MA, USA) and were Sanger-sequenced by The Central Laboratory of High-Performance Technologies (LACTAD, Campinas, Brazil). The obtained sequence was submitted to GenBank, and the following accession number was assigned: BK068764.

To determine whether the A301S mutation is associated with gender in *E. heros*, 30 male and 30 female stink bugs were separated into different containers, frozen for 15 min at −20 °C and had their DNA extracted. The primers used in qPCR assays (Table 1) were applied to amplify the fragment of the *Rdl* gene containing the A301S mutation, using PowerUp SYBR Green Master Mix (Applied Biosystems) to assess whether gene copy number differed between males and females.

### 2.5. qPCR Assays to Monitor A301S Mutation in E. heros

Quantitative real-time PCR (qPCR) to detect the A301S mutation in *E. heros* was performed using 1 μL of DNA (50 ng/μL), 5 μL of TaqMan Genotyping Master Mix (Applied Biosystems™, Waltham, MA, USA), 0.7 μL of 10 μM primers (forward and reverse), 0.2 μL of 10 ng/μL probe and nuclease-free water to a final reaction volume of 10 μL. Primers and probes (Table 1) were designed using Geneious Prime software v.2023.2.1 (Biomatters Ltd., Auckland, New Zealand). The probes contained different fluorescent dyes and were used for the single-allele detection of wildtype (A301) and mutant (S301) gene fragments in a modified real-time PCR assay. The amplification reactions were conducted at 95 °C for 10 min (1×), followed by 40 cycles at 95 °C for 15 s and 60 °C for 60 s using a QuantStudio 6 Flex Real-Time PCR (Applied Biosystems™, Waltham, MA, USA). To identify whether the A301S mutation is related to gender in *E. heros*, 2 μL of DNA (20 ng/μL), 5 μL of PowerUp SYBR Green Master Mix (Applied Biosystems), 0.4 μL of 10 μM primers (forward and reverse) and nuclease-free water for a final reaction volume of 10 μL were added. The amplification reactions were conducted at 98 °C for 3 min (1×), followed by 40 cycles at 98 °C for 15 s and 60 °C for 30 s using a QuantStudio 6 Flex Real-Time PCR (Applied Biosystems™, Waltham, MA, USA). The resistance allele frequency detected in the PCR-based allelic discrimination assay was correlated with the survivorship observed in phenotypic ethiprole vial test monitoring. Statistical analysis was conducted using GraphPad Prism 9 (GraphPad Software, San Diego, CA, USA).

### 2.6. Statistical Analysis of Genetic Diversity and Population Differentiation

A genetic diversity analysis was performed for the A301S mutation to detect spatial and temporal changes in *E. heros* populations sampled in different cropping seasons. Observed heterozygosity (Ho) and expected heterozygosity (He) were estimated, and the locus was tested for Hardy–Weinberg equilibrium using the HardyWeinberg package in R software (https://www.r-project.org, doi:10.18637/jss.v064.i03, accessed on 1 December 2024). This analysis was conducted by treating each sampled location as an individual population and by considering all samples as part of a single unified population.

## 3. Results

### 3.1. Phenotypic Resistance Monitoring of E. heros Using the Label-Recommended Dose of Ethiprole

**Figure 2:**
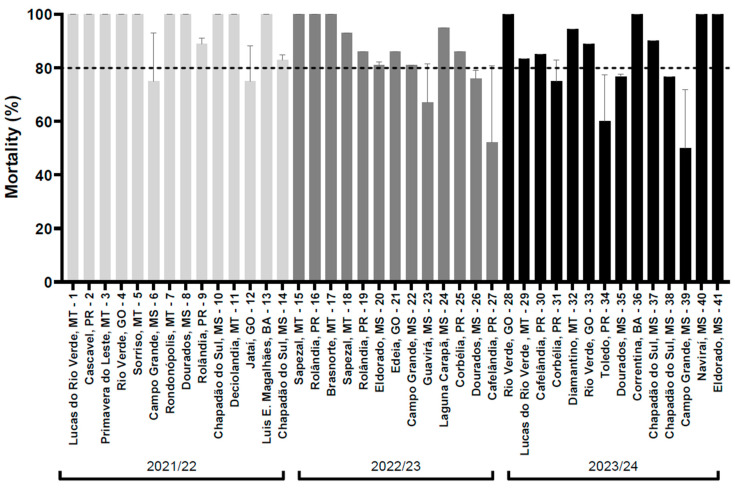
Susceptibility of 41 *E. heros* populations to the field label dose (150 g a.i./ha) of ethiprole in adult vial bioassays performed during the 2021/22, 2022/23 and 2023/24 soybean seasons in Brazil. Mortality assessment was performed after 24 h of continuous exposure to ethereal.

To monitor the efficacy of ethiprole against *E. heros* collected from soybean-growing regions in the states of Bahia (BA), Goiás (GO), Mato Grosso (MT), Mato Grosso do Sul (MS) and Paraná (PR) in Brazil, we conducted adult vial bioassays to assess phenotypic resistance over three crop seasons (2021/22, 2022/23 and 2023/24). Throughout these seasons, most field populations exhibited high mortality rates (>80%) when exposed to the field label dose of ethiprole (150 g a.i./ha) in contact bioassays (Figure 2). Mortality scores ranged from 75% to 100% in 2021/22, 52% to 100% in 2022/23 and 50% to 100% in 2023/24. Of the 41 *E. heros* populations evaluated, only 4 showed mortality rates below 70%: Guavirá/MS (67%) and Cafelândia/PR (52%) in 2022/23, and Toledo/PR (60%) and Campo Grande/MS (50%) in 2023/24. Our results indicated no significant shift in the susceptibility of *E. heros* to ethiprole between 2021 and 2023/24 (*p*-value = 0.7946, one-way ANOVA followed by Tukey’s post hoc test).

### 3.2. RDL-GABA-Gated Chloride Channel Partial Sequencing

**Figure 3:**
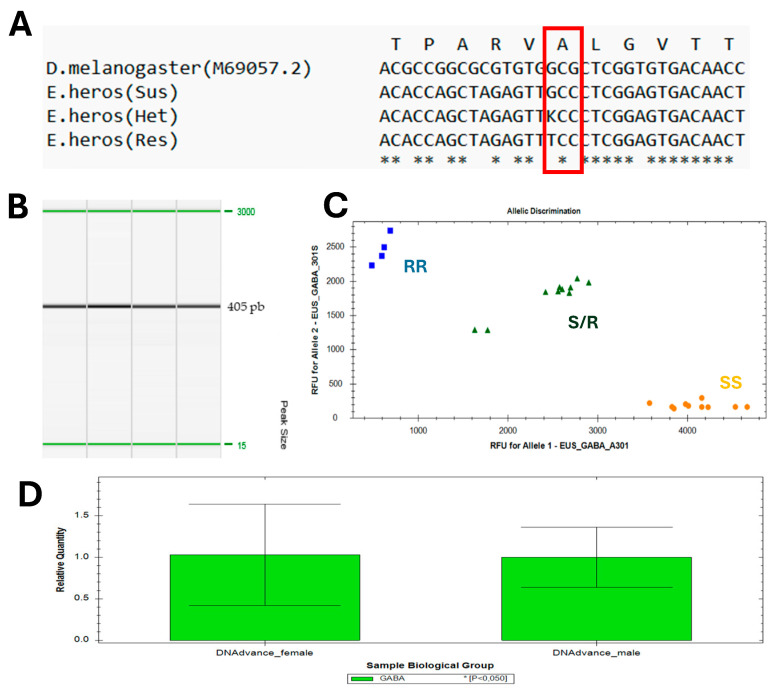
A301S mutation detected in RDL-GABA-gated chloride channel in *E. heros*. (**A**) Alignment of the nucleic acids and amino acid sequences of a partial region of the RDL-GABA receptor, targeting the A301S mutation in the *D. melanogaster* RDL-GABA receptor sequence (GenBank M69057.2), compared to *E. heros* susceptible (Sus), heterozygote (Het) and resistant (Res) genotypes. Consensus = *. (**B**) PCR amplicons showing a size of 405 bp between the alignment markers (15–3000 bp). (**C**) qPCR TaqMan assay for detecting the A301S mutation in *E. heros* samples. The x-axis represents fluorescence measurements for the VIC probe, which detects the G allele, while the y-axis represents fluorescence measurements for the FAM probe, which detects the T allele. The three clusters correspond to resistant (RR), heterozygous (S/R) and susceptible (SS) genotypes. (**D**) Graphic showing no difference in relative quantity of *Rdl*-GABA gene copy number in male and female Neotropical brown stink bugs via q-PCR.

To investigate the presence of mutations potentially conferring target-site resistance to ethiprole, we amplified and sequenced a partial transmembrane fragment (domains M1-M3) of the RDL-GABA-gated chloride channel—the target site of ethiprole. The obtained fragments of 405 bp (Figure 3A,B) showed high similarity at the amino acid level when compared to *D. melanogaster Rdl* (GenBank reference M69057.2). A total of 2692 *E. heros* adults were analyzed over three seasons. Several synonymous single-nucleotide polymorphisms (SNPs) were detected, but the only non-synonymous SNP identified was at position A301 (*Drosophila* numbering), corresponding to position A270 in *E. heros*, located in transmembrane domain II. This mutation resulted in the substitution of the amino acid alanine (GCC) by serine (TCC), known to confer target-site resistance (Figure 3A). Additionally, a TaqMan genotyping assay was developed to detect the G-to-T substitution that results in the alanine-301-serine (A301S) mutation in *E. heros* (Figure 3C). This assay identified three distinct genotype clusters: RR (resistant), S/R (heterozygous) and SS (susceptible). Finally, the 129 bp fragment was equally amplified in both sexes, as shown in Figure 3D. As no difference in *Rdl* gene copy number was observed between male and female insects, it was concluded that the A301S mutation is not related to gender in *E. heros*.

### 3.3. Genotyping of E. heros Samples

**Figure 4:**
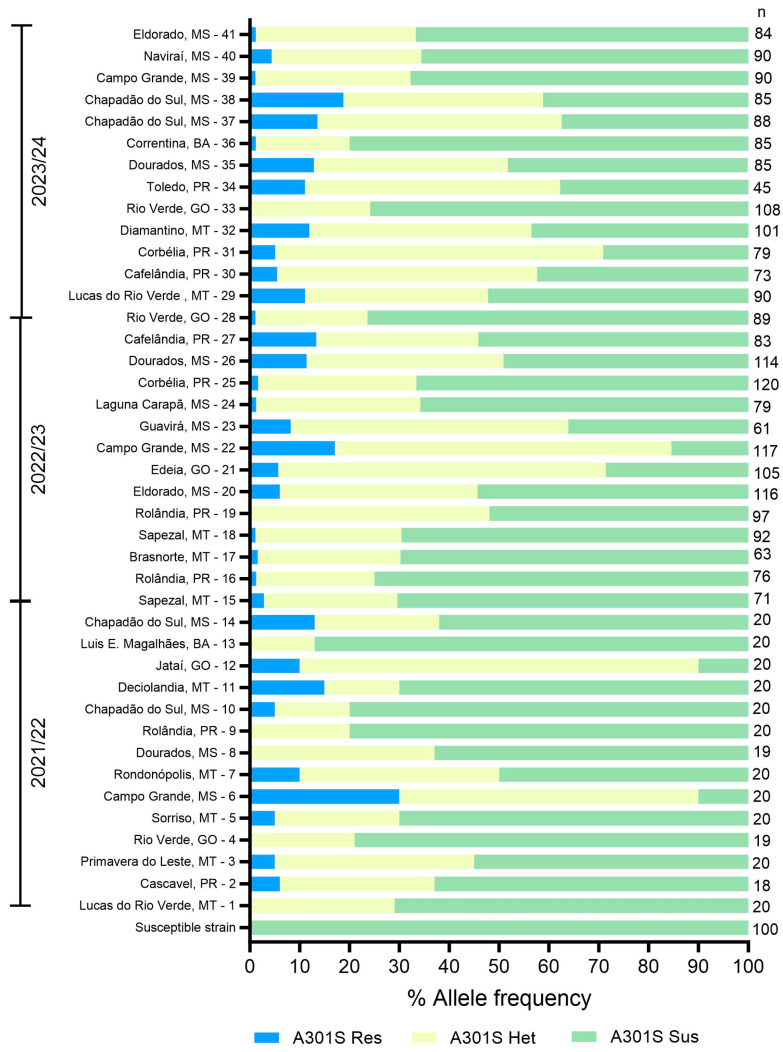
Allele frequency of A301S mutation monitored in *E. heros* throughout soybean crop seasons 2021/22, 2022/23 and 2023/24 in BA, GO, MS, MT and PR states in Brazil. Legend: blue represents A301S homozygous resistant (Res) genotypes, yellow represents heterozygous (Het) genotypes, and green represents homozygous susceptible (Sus) genotypes. The number on the right side indicates the quantity of insects genotypically analyzed in each population.

After confirming the presence of the A301S mutation in *E. heros* for the first time, we subsequently monitored its allele frequency across seasons and assessed its impact on ethiprole efficacy. To achieve this, a total of 2692 insects from 41 *E. heros* populations were analyzed using the TaqMan qPCR assay. Figure 4 shows the allele frequency of the A301S mutation in *E. heros* populations from the 2021/22 to 2023/24 soybean seasons in Brazil. Collection dates and the number of insects analyzed in the genotyping assays are provided in Table S1.

Genotyping data revealed that the A301S resistance allele consistently appeared at low frequencies across all populations sampled between 2021 and 2024, with the highest frequency observed at 31.1% in Campo Grande/MS (6) during the 2021/22 season. The states of Mato Grosso do Sul (MS) and Paraná (PR) recorded the highest resistance genotype frequencies, with maximum rates of 13.3% and 18.8%, respectively, over the seasons. Additionally, no increase in the frequency of the resistant genotype allele was observed over the three seasons.

Some populations from the same city were collected two times within the same season (Chapadão do Sul/MS in 2021/22, Rolândia/PR in 2022/23 and Chapadão do Sul/MS again in 2023/24), but no significant changes in the frequency of the resistant genotype were observed. Additionally, Campo Grande/MT was the only location where insects were collected across all three seasons (samples 6, 22 and 39), and a reduction in the frequency of the resistant genotype was noted over time. Homozygous resistant genotypes were absent in only 7 out of the 41 analyzed populations (1, 4, 8, 9, 13, 19 and 33).

The heterozygous genotype was present in all populations, with frequencies ranging from 13% to 18.8% in BA, 15% to 44.6% in MT, 20% to 65.8% in PR, 15% to 67.5% in MS and 21% to 80% in GO. Therefore, at least one allele of the A301S mutation was detected in every population evaluated in this study, despite the low frequency of the resistant genotype, possibly indicating fitness costs associated with resistant alleles in *E. heros.*

**Figure 5:**
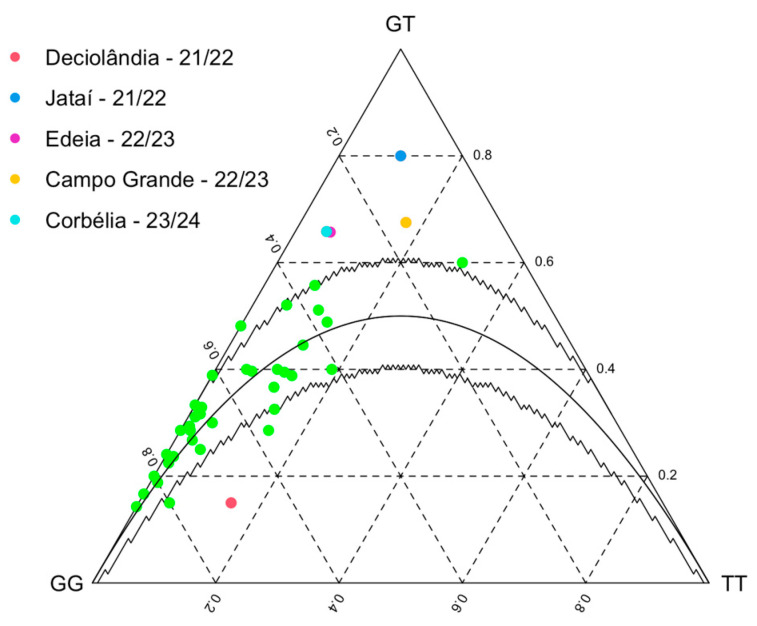
Ternary plot of A301S mutation in 41 populations of *E. heros* collected in Brazil. The acceptance region of Haldane’s exact test is shown, and green dots within this region represent populations in equilibrium, whereas differently colored points represent populations that are significantly outside Hardy–Weinberg equilibrium.

To accurately assess the frequency of susceptibility and resistance in field populations, we specifically analyzed the A301S mutation associated with resistance at the nucleotide level, considering allele frequencies across all samples for the *Rdl* gene at each sampling location. The polymorphic codon position deviated from Hardy–Weinberg equilibrium in only five sampled locations. In four locations (Jataí, Edéia, Campo Grande and Corbélia), the observed heterozygosity exceeded expectations, while in Deciolândia, it was lower than expected (Table 2 and Figure 5). Notably, this heterozygote disequilibrium was not consistently observed across the three seasons in the same regions in Brazil.

### 3.4. Relationship Between Ethiprole Efficacy and Resistance Allele Frequency in E. heros

**Figure 6:**
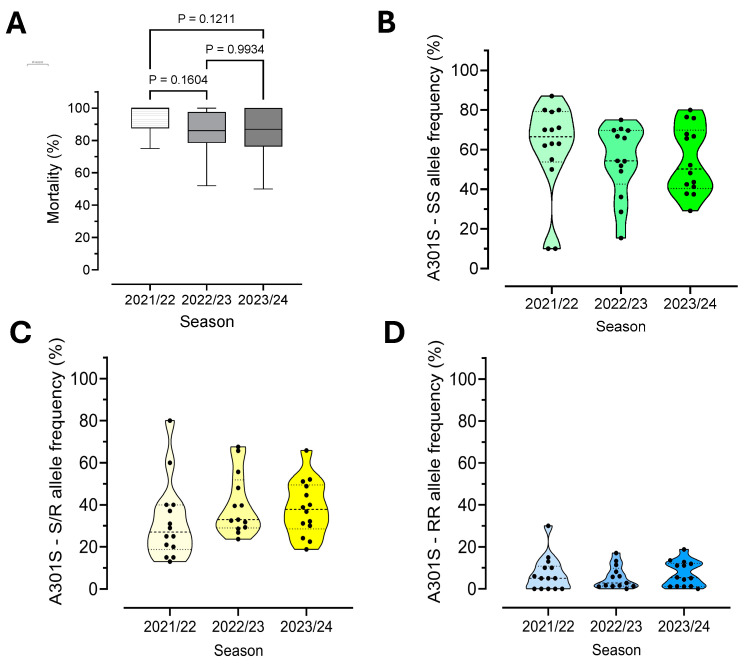
Phenotyping and genotyping assays in *E. heros* sampled across three seasons. (**A**) Efficacy of ethiprole field label dose (vial test, 150 g a.i./ha, 24 h of exposure) against 41 *E. heros* field populations. Violin plots showing the susceptible (SS) (**B**), heterozygous (S/R) and (**C**) and resistant (RR) (**D**) allele frequency (%) of each population across the seasons. Points in the graph represent individual populations. Genotype frequency was not significantly different between seasons.

Most field populations collected during the 2021/22, 2022/23 and 2023/24 cropping seasons exhibited high mortality rates (>80%) when exposed to the field label dose of ethiprole (150 g a.i./ha) (Figure 6A). However, a decline in mortality was observed in the last two seasons compared to the first, but differences between seasons were not significant. Over the seasons, the wildtype and A301S heterozygous genotype frequencies showed some variation but did not significantly differ across all populations analyzed, while the homozygous resistant (RR) genotype remained stable at a low frequency (around 20%) (Figure 6B–D).

**Figure 7:**
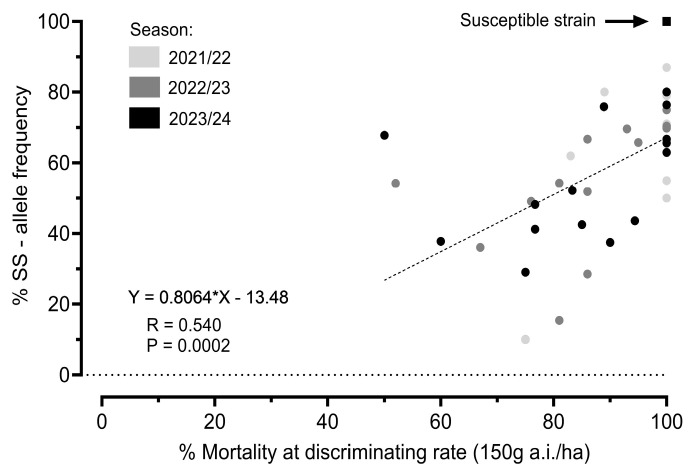
Linear regression analysis revealing a significant correlation (*p* = 0.0002) between RDL-GABA receptor resistance allele frequency (A301S) and the survival of field-collected *E. heros* populations at a discriminating rate of ethiprole (vial test, 150 g a.i./ha).

A linear regression analysis showed that the mortality rates from the large-scale phenotypic screening using the field label dose of ethiprole (150 g a.i./ha) were significantly correlated with a decreasing abundance of susceptible alleles, as determined by the PCR-based allelic discrimination assay (*p*-value = 0.0002) (Figure 7).

Our data indicated that the reduction in ethiprole susceptibility observed in adult vial tests over the seasons is linked to an increasing frequency of the heterozygous genotype, suggesting that the presence of even one A301S mutant allele slightly affects ethiprole efficacy.

### 3.5. Genotyping of E. heros Survivors of Vial Bioassays

**Figure 8:**
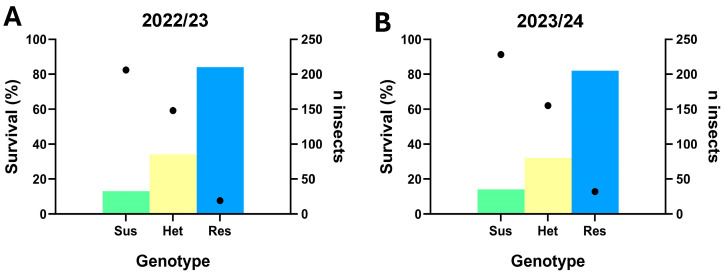
Correlation between ethiprole efficacy and the presence of A301S genotypes in survivors out of (**A**) 373 *E. heros* adults from 13 populations collected during the 2022/23 season and (**B**) 413 adults from 14 populations collected in 2023/24. Bars represent the survival rate (%) of each genotype in adult vial bioassays (150 g a.i./ha) 24 h after ethiprole exposure, while the dark dots indicate the number of insects for the specific genotype (Sus—susceptible; Het—heterozygous; Res—resistant).

In the 2022/23 season, 373 insects from 13 populations were evaluated for susceptibility at the label dose of ethiprole (150 g a.i./ha) in vial tests, including 206 scored as susceptible, 148 as heterozygous and 19 as resistant homozygous genotypes. These sample groups exhibited adult vial test survival rates of 13%, 34% and 84%, respectively (Figure 8A). Similar results were observed in the 2023/24 season, when 415 insects from 14 populations were assessed, with 228 showing the susceptible genotype, 155 the heterozygous genotype and 32 the homozygous resistant genotype. Survival rates in adult vial tests were 14%, 32% and 82%, respectively (Figure 8B).

Our data revealed that homozygous resistant genotypes largely survived (around 84%) exposure to the label dose of ethiprole (150 g a.i./ha) in adult vial bioassays. In contrast, the susceptible and heterozygous genotypes exhibited survival rates of 12 and 32%, respectively, possibly indicating an incompletely recessive trait.

## 4. Discussion

The present resistance monitoring study, investigating 41 NBSB populations sampled in geographically distant Brazilian soybean fields, revealed the presence of the A301S mutation in *E. heros* RDL-GABA receptors commonly known to confer resistance to fiprole insecticides such as ethiprole, a non-competitive GABA-gated chloride channel antagonist registered for stink bug control in various field crops in Brazil. Indeed, the detected A301S mutation is the most frequent amino acid substitution conferring target-site resistance in the RDL-GABA receptor [26], and was previously described to affect the binding of IRAC MoA group 2 insecticides to GABA-gated chloride channels in several insect pests [22,26]. This mutation was first discovered in *D. melanogaster*, causing 4000-fold resistance to dieldrin [23] and moderate resistance to phenylpyrazoles [29]. The mutation was also found in other hemipteran pests such as *Nilaparvata lugens* and *Sogatella furcifera*, where it was linked to rather low levels of resistance to fipronil [26,30,34] but significantly higher levels of ethiprole resistance [27]. Electrophysiological studies with functionally expressed *N. lugens* RDL-GABA receptors confirmed that the A301S mutation affects ethiprole binding to a significantly higher extent than fipronil binding [27]. However, the impact of this mutation varies among pest species. Almost no impact on fiprole insecticide efficacy has been shown after its introduction into *Plutella xylostella* and in subsequent bioassays with transgenic larvae [35], or in functionally expressed mutant RDL-GABA receptors of *Spodoptera litura* [36]. Here, we confirmed the presence of the A301S mutation in *E. heros* RDL-GABA receptors and demonstrated its correlation with lower ethiprole efficacy in phenotypic assays, but further in vivo and in vitro studies at the dose–response level are warranted to quantitatively investigate the impact of this mutation on ethiprole resistance in isogenic *E. heros* strains and functionally expressed RDL-GABA receptors, respectively.

We showed that during the soybean seasons 2021/22, 2022/23 and 2023/24, most of the field-collected populations showed high mortality (>80%) when exposed to the field label dose of ethiprole (150 g a.i./ha) in adult vial bioassays, thus largely confirming previous results that also showed >80% efficacy of recommended label rates of ethiprole against *E. heros* field samples collected in 2020/21 and tested in laboratory assays [15]. However, in our study, a non-significant decline in ethiprole efficacy was observed in the last two seasons when compared to the first season, 2021/22, likely due to an increased frequency of heterozygotes while the frequency of homozygotes remained rather stable. The correlation between genotyping and phenotyping data suggests that even the presence of a single A301S mutant allele can reduce the efficacy of ethiprole, raising concerns about a consistent, albeit slow emergence of homozygous resistant individuals from mating events between heterozygous individuals surviving recommended label rates. Notably, *E. heros* genotypes homozygous for A301S largely survived the label dose of ethiprole (84% survival), but the mean frequency of the resistant genotypes remained low (6.7%) and stable across all three seasons, helping to mitigate the risk of widespread resistance. The fact that most heterozygotes showed a low survival rate in ethiprole discriminating dose bioassays suggests an incompletely recessive trait, although we cannot rule out the presence of and selection for additional mechanisms of resistance, as demonstrated in *N. lugens* with the overexpression of paralogous P450s (CYP6ER1v) conferring metabolic ethiprole resistance [37].

Studies in *D. melanogaster* revealed semidominant genetics for the A301S trait [38,39]; however, future reciprocal crossing studies with A301S isogenic homozygotes and wildtype strains will help to more clearly define the genetics of *Rdl*-GABA mediated target-site resistance to ethiprole in *E. heros*. It is tempting to speculate that the low frequency of the homozygous resistant genotype in NBSB field populations is possibly linked to fitness costs in the absence of ethiprole and is affecting their survival in the field. Some authors suggested that severe functional constraints in nature are expected in individuals with mutations associated with resistance, especially if essential genes, e.g., ion channel subunits such as *Rdl*, are affected by mutations affecting the physiology of the pest under adverse environmental conditions [40]. Amino acid substitutions in the *Drosophila* RDL-GABA receptor conferring cyclodiene resistance have been shown to be associated with a temperature-sensitive phenotype, where resistant flies exhibited temporary paralysis when exposed to high temperatures and were unable to move, compared to cyclodiene-susceptible flies [24]. However, the effect of temperature-sensitive paralysis linked to *Rdl* mutations has only been investigated in the laboratory and not under applied conditions. Future studies are necessary to investigate whether the same sensitivity toward high temperatures observed in *D. melanogaster* might impact the overall fitness of pests such as *E. heros,* as this would likely affect the presence and survival of ethiprole-resistant homozygotes in the field.

NBSB resistance management largely relies on the availability of diverse chemical classes of insecticides with different MoAs applied in rotation following an MoA treatment window approach, where a treatment window is defined by the lifecycles of the pest insect [32]. Among the insecticides available for NBSB control, ethiprole showed strong performance in both field and laboratory assays [16,31], most likely due to the low frequency of (homozygous) resistance alleles as shown here. Insecticide resistance in *E. heros* has already been reported for several other chemical classes, including cyclodienes (endosulfan, IRAC MoA group 2A), organophosphates (monocrotophos and methamidophos, IRAC MoA group 1B), neonicotinoids (imidacloprid and thiamethoxam, IRAC MoA group 4A) and pyrethroids (IRAC MoA group 3A) such as bifenthrin, λ-cyhalothrin and β-cyfluthrin [9,10,11,12,13,14]. To mitigate resistance development, rotating insecticides with different modes of action is essential to reduce selection pressure, especially on those chemical classes with a long history in stink bug control [32,33]. Applications of insecticides targeting RDL-GABA receptors in NBSB have a history in Brazil, because endosulfan has been used for decades to control this pest and resistance has been reported as far back as in the late 1990s [11,41], although the mechanisms of endosulfan resistance in *E. heros* have remained elusive, whereas endosulfan resistance based on the A301S target-site mutation has been functionally confirmed in other insects, e.g., in *Drosophila* lines expressing a mutant RDL-GABA receptor [42]. In light of the results presented here, it seems fair to assume that the A301S resistance allele was already present in NBSB field populations when ethiprole was launched in 2021 due to previous selection pressure by endosulfan treatments.

Resistance management strategies based on chemical insecticides rely on the efficacy of registered insecticides and the absence of cross-resistance issues between chemical classes; therefore, it is important to investigate the molecular mechanisms of resistance. However, molecular mechanisms of resistance have not been unveiled yet for any of the chemical classes used to control *E. heros*, except for ethiprole (this study). Studies have shown that *E. heros* populations in Brazil with decreased pyrethroid susceptibility showed an overexpression of detoxification enzymes like cytochrome P450 monooxygenases, glutathione S-transferases and esterases [9,41], but individual genes or enzymes have not yet been identified and functionally validated. The involvement of metabolic enzymes has been predicted based on bioassays with synergistic compounds known to inhibit detoxification enzymes. Boff et al. [43] tested nano-encapsulated formulations of bifenthrin and λ-cyhalothrin with piperonyl butoxide (a P450 inhibitor) and dietylmaleimide (a GSH depletion agent), and demonstrated their potential to improve the control of *E. heros* with low pyrethroid susceptibility. Such synergist bioassays could also help to determine if mechanisms of metabolic resistance contribute to lower ethiprole susceptibility in *E. heros* populations.

Ethiprole has only recently been introduced as an additional mode of action for *E. heros* control in Brazil, and its efficacy should be preserved by appropriate resistance management strategies, including regular monitoring campaigns to follow the frequency and spread of the resistance allele in *E. heros* across a broad geographic range, particularly considering the fact that *E. heros* shows limited spatial dispersal [44,45,46], so that control measures may be adopted at a local scale. However, we strongly recommend the implementation of resistance management strategies utilizing the entire available range of insecticide MoAs registered. This will reduce selection pressure, delaying the evolution of resistance in *E. heros* and helping to conserve the efficacy of the limited arsenal of insecticide modes of action for sustainable stink bug management in Brazil.

## 5. Conclusions

In conclusion, ethiprole resistance in *E. heros* was detected for the first time and linked to the presence of an A301S mutation in RDL-GABA receptors. *E. heros* field populations are dominated by homozygous susceptible and heterozygous genotypes largely susceptible to recommended label rates of ethiprole in vial bioassays. In contrast, homozygous resistant genotypes largely survived when exposed to ethiprole in discriminating dose bioassays, underpinning the importance of the implementation of resistance management strategies. We developed a genotyping diagnostic tool based on TaqMan assays to monitor the frequency and spread of RDL-GABA target-site resistance as a foundation for an effective resistance management program, covering not just ethiprole but also other important modes of action, for sustainable Neotropical brown stink bug control in the future.

## Figures and Tables

**Table 1:** Primers and probes used to detect RDL-A301S mutation in *E. heros* by Sanger sequencing and qPCR assays.

	Primer	Sequence (5′–3′)	Size (bp)
PCR/Sanger	Eh—PF	ATAAGGGTAATGGAGACGG	405
	Eh—PR	AACTAGCAAAGGAGAAAAGG	
qPCR	A301S Eh—PF	CGGGCTCATCGTCATCATCA	20
	A301S Eh—PR	GCGGCGTTAGTAGATGACATG	21
	Sus A301S Eh	5′-[VIC]AGTTGCCCTCGGAGTG[MGBNFQ]-3′	16
	Res A301S Eh	5′-[FAM]AGTTTCCCTCGGAGTG[MGBNFQ]-3′	16

**Table 2:** Hardy–Weinberg equilibrium test results for the five populations of *E. heros* with an excess or lack of heterozygotes (*p*-value < 0.05) for A301S mutation positions related to the determination of susceptibility and resistance. Ho = observed heterozygosity; He = expected heterozygosity; *p*-value = Haldane’s exact test.

Season	Population	Ho	He	*p*-Value
2021/22	Deciolândia—MS	0.150	0.358	0.011
	Jataí—GO	0.800	0.513	0.007
2022/23	Edeia—GO	0.657	0.476	0.000
	Campo Grande—MS	0.657	0.502	0.000
2023/24	Corbélia—PR	0.658	0.474	0.000

## Data Availability

The original contributions presented in this study are included in the article/Supplementary Material. Further inquiries can be directed to the corresponding authors. The partial *Rdl* nucleotide sequences reported in this study have been deposited in GenBank and are available under the accession number BK068764.

## Supplementary Materials

The following supporting information can be downloaded at https://www.mdpi.com/article/10.3390/insects16040422/s1. Table S1: *Euschistus heros* populations collected from different Brazilian locations in three crop seasons (2021/22, 2022/23 and 2023/24) for monitoring and characterizing susceptibility to ethiprole (vial test).
