# Supplementary material for: The Frequency and Spread of a GABA-Gated Chloride Channel Target-Site Mutation and Its Impact on the Efficacy of Ethiprole Against Neotropical Brown Stink Bug, Euschistus heros (Hemiptera: Pentatomidae)
Source: Insects. 2025 Apr 17;16(4):422. doi: 10.3390/insects16040422 (PMC12028171; doi:10.3390/insects16040422)
Supplement: Supplementary file 1 [file insects-16-00422-s001.zip › insects-3549135-supplementary.pdf]

**Table S1** – *Euschistus heros* populations collected from different Brazilian locations in three crop seasons (2021/22, 2022/23 and 2023/24) for monitoring and characterizing susceptibility to ethiprole (vial test).

| Crop Season | Population | City | State | Latitude | Longitude | Collect Date |
|---|---|---|---|---|---|---|
| **Vial test** | | | | | | |
| | Susceptible strain | Paulínia | SP | -22,7617 | -47,1541 | 01/01/2013 |
| 2021/22 | 1 | Lucas do Rio Verde | MT | -13,0063 | -55,9680 | 26/01/2022 |
| | 2 | Cascavel | PR | -25,0561 | -53,6427 | 25/01/2022 |
| | 3 | Primavera do Leste | MT | -15,3109 | -54,2423 | 15/02/2022 |
| | 4 | Rio Verde | GO | -17,4164 | -51,7426 | 18/02/2022 |
| | 5 | Sorriso | MT | -12,4264 | -55,6377 | 23/02/2022 |
| | 6 | Campo Grande | MS | -20,7077 | -54,7756 | 25/02/2022 |
| | 7 | Rondópolis | MT | -17,1619 | -54,7537 | 22/02/2022 |
| | 8 | Dourados | MS | -22,2354 | -54,9943 | 25/02/2022 |
| | 9 | Rolândia | PR | -23,3105 | -51,3695 | 07/03/2022 |
| | 10 | Chapadão do Sul | MS | -18,7799 | -52,6480 | 04/03/2022 |
| | 11 | Deciolândia | MT | -14,1833 | -51,5534 | 08/03/2022 |
| | 12 | Jataí | GO | -17,8784 | -51,7204 | 24/03/2022 |
| | 13 | Luis Eduardo Magalhães | BA | -12,1233 | -46,0258 | 06/04/2022 |
| | 14 | Chapadão do Sul | MS | -18,7722 | -52,6446 | 12/04/2022 |
| 2022/23 | 15 | Sapezal | MT | -13,5082 | -58,7941 | 06/02/2023 |
| | 16 | Rolândia | PR | -23,2705 | -51,4845 | 08/02/2023 |
| | 17 | Brasnorte | MT | -12,7947 | -58,0892 | 10/02/2023 |
| | 18 | Sapezal | MT | -13,5082 | -58,7941 | 17/02/2023 |
| | 19 | Rolândia | PR | -23,2705 | -51,4845 | 08/03/2023 |
| | 20 | Eldorado | MS | -23,7741 | -54,2800 | 08/03/2023 |
| | 21 | Edeia | GO | -17,3381 | -49,9344 | 09/03/2023 |
| | 22 | Campo Grande | MS | -20,3119 | -54,5364 | 09/03/2023 |
| | 23 | Guavirá | MS | -20,4888 | -56,5833 | 09/03/2023 |
| | 24 | Laguna Carapã | MS | -22,7088 | -55,1226 | 10/03/2023 |
| | 25 | Corbélia | PR | -24,7996 | -53,2965 | 13/03/2023 |
| | 26 | Dourados | MS | -22,2788 | -54,6495 | 15/03/2023 |
| | 27 | Cafelândia | PR | -24,6180 | -53,2971 | 26/03/2023 |
| 2023/24 | 28 | Rio Verde | GO | -17,4578 | -50,9305 | 12/01/2024 |
| | 29 | Lucas do Rio Verde | MT | -13,0069 | -55,9686 | 25/01/2024 |
| | 30 | Cafelândia | PR | -24,5868 | -53,3464 | 01/02/2024 |
| | 31 | Corbélia | PR | -24,7792 | -53,2646 | 01/02/2024 |
| | 32 | Diamantino | MT | -14,1846 | -57,5540 | 15/02/2024 |
| | 33 | Rio Verde | GO | -17,7873 | -50,9978 | 17/02/2024 |
| | 34 | Toledo | PR | -24,7258 | -53,7681 | 01/03/2024 |
| | 35 | Dourados | MS | -22,3010 | -54,6206 | 06/03/2024 |
| | 36 | Correntina | BA | -13,6511 | -45,2601 | 15/03/2024 |
| | 37 | Chapadão do Sul | MS | -18,7740 | -52,6485 | 22/03/2024 |
| | 38 | Chapadão do Sul | MS | -18,7740 | -52,6485 | 09/04/2024 |
| | 39 | Campo Grande | MS | -20,6504 | -54,6273 | 17/04/2024 |
| | 40 | Naviraí | MS | -23,0513 | -54,2388 | 17/04/2024 |
| | 41 | Eldorado | MS | -23,7557 | -54,2716 | 25/04/2024 |
